# Antifungal Activity of Aqueous Extracts of Some Dominant Algerian Medicinal Plants

**DOI:** 10.1155/2017/7526291

**Published:** 2017-10-31

**Authors:** Nasrine Salhi, Sultan Ayesh Mohammed Saghir, Valeria Terzi, Iman Brahmi, Naima Ghedairi, Samia Bissati

**Affiliations:** ^1^Laboratoire de Bio-Ressources Sahariennes: Préservation et Valorisation, Faculté des Sciences de la Nature et de la Vie, Université Kasdi Merbah Ouargla, Ouargla 30000, Algeria; ^2^School of Pharmaceutical Sciences, Pharmacology Department, Universiti Sains Malaysia, 11800 Penang, Malaysia; ^3^CREA-GB, Research Centre for Genomics and Bioinformatics, Via San Protaso 302, Fiorenzuola d'Arda, 29017 Piacenza, Italy; ^4^Faculté des Sciences de la Nature et de la Vie, Université Kasdi Merbah Ouargla, Ouargla 30000, Algeria

## Abstract

**Aim:**

This study investigated the antifungal properties of aqueous extracts obtained from indigenous plants that grow spontaneously in the Northern Sahara of Algeria. The activities of these plants in controlling two fungal species that belong to* Fusarium* genus were evaluated in an in vitro assay.

**Materials and Methods:**

Fresh aerial parts of four plant species (*Artemisia herba alba, Cotula cinerea, Asphodelus tenuifolius*, and* Euphorbia guyoniana*) were collected for the preparation of aqueous extracts. Two levels of dilution (10% and 20%) of the pure extracts were evaluated against* Fusarium graminearum* and* Fusarium sporotrichioides*.

**Results:**

The results of this study revealed that the* A. herba alba*,* C. cinerea, A. tenuifolius*, and* E. guyoniana* aqueous extracts are effective at both concentrations of 10% and 20% for the* Fusarium mycelia* growth inhibition. In particular,* A. tenuifolius* extract is effective against* F. graminearum*, whereas* F. sporotrichioides* mycelium growth is strongly affected by the* E. guyoniana* 20% extract. The phytochemical characterization of the compositions of the aqueous extracts has revealed that the presence of some chemical compounds (tannins, flavonoids, saponins, steroids, and alkaloids) is likely to be responsible for the antifungal activities sought.

**Conclusion:**

The antifungal properties of* A. herba alba*,* C. cinerea*,* A. tenuifolius*, and* E. guyoniana* make these plants of potential interest for the control of fungi affecting both wheat yield and safety.

## 1. Introduction

It is commonly recognized that several classes of phytopathogenic fungi can cause relevant yield losses of cereals. Moreover, phytopathogenic can affect cereal grains during storage, rendering them unfit for human consumption by lowering the quality and safety of the derived products. The establishment of fungal infections in cereals has therefore several consequences, ranging from yield lowering to retarding their nutritive value until the contamination of grains with dangerous mycotoxins [1]. The growth of phytopathogenic fungi in crops is also responsible for the off-flavor formation and production of allergenic compounds [2, 3].* Aspergillus*,* Fusarium*, and* Penicillium* species are the most important fungi affecting the yield of small grain cereal and causing spoilage of the derived foodstuffs. Various strategies can be employed for the control of fungal infection, ranging from the adoption of specific agronomic practices to the development of resistant varieties [4]. The chemical control remains now one of the major measures that can be implemented for the reduction of the incidence of plant disease. To this aim, over the past years, numerous chemical pesticides such as benzimidazoles, imazalil, organic, and inorganic sulfur compounds and oxidizing materials have been introduced to control the plant disease. However, the current concern has been raised about their extensive use because of the potential environmental problems, toxicity to humans, establishment of fungal resistant races, and sometimes high costs of such combinations. Recently, it has been pointed out that over 200 species of plant pathogens are resistant to chemical pesticides and most of these pesticides have various side effects [5]. For these reasons, searching for naturally occurring potential antifungal and antimicrobial agents to be used for crop protection and food preservation is paramount and takes much attention, even because of the awareness of natural and biological food products [6]. Therefore, despite the facts that the use of natural products for crop protection is not new and that it was adopted ever since man has taken to farming, it has now been rediscovered, with considerable research for biocides that are environmentally safe and easily biodegradable [6–8].

For plants to protect them against pathogens, they produce and exude myriad of secondary metabolites, which play important roles as defense mechanisms against various organisms. Two main classes of such compounds can be identified: the constitutive and inducible ones. The first compounds are present even in healthy plants, whereas the second classes of metabolites are synthesized only in response to pathogen attack. The first class of metabolites includes phenols (such as flavonoids, tannins, and alkaloids) that can be found in most of the essential oils that can be extracted from aromatic and medicinal plants. Moreover, those constitutive compounds can be categorized in “quantitative” compounds and “qualitative” compounds. While the “quantitative” compounds include tannins, phenols, flavonoids, and terpenes that are characterized by high molecular weight and low toxicity, the “qualitative” compounds include alkaloids, triterpenes, naphthoquinones, and anthraquinones that are characterized by low molecular weights, high toxicity, and strong biological activities [9].

In several researches, extracts of various higher plants have been reported to exhibit antifungal properties under* in vitro* laboratory trials [10–15]. In particular, wild plants seem to be a promising source that can possess useful metabolites. Thus, this study was designed to explore the* in vitro* potential antifungal activity of aqueous extracts of the aerial parts of four dominant Algerian medicinal plants which are* Artemisia herba alba, Cotula cinerea, Asphodelus tenuifolius*, and* Euphorbia guyoniana*. The efficacy and potency of the extracts of these plants were evaluated against the cereal pathogens* Fusarium graminearum* and* Fusarium sporotrichioides*. The essential oils of these plants have been already characterized for their chemical composition and biological effects, mainly against human and animal pathogens [16–18]. In comparison to previous research, one of the most innovative aspects of this study is the evaluation of the effects of the Algerian medicinal plants against agriculturally important cereal pathogens, starting from aqueous extract that can be prepared at room temperature through a simple and low cost infusion step.

## 2. Materials and Methods

### 2.1. Plant Collection

The fresh aerial parts of* Artemisia herba alba, Cotula cinerea*,* Asphodelus tenuifolius*, and* Euphorbia guyoniana* were collected during the vegetative stage in Algerian natural habitats. The samples were air-dried, grinded in a Wiley Mill to fine uniform texture, and stored in glass jars until use.

### 2.2. Preparation of the Plant Aqueous Extracts

First, stock aqueous extracts were obtained by soaking 10 g of air-dried and milled plant material in 100 ml of distilled water (10% w/v) at room temperature (20 ± 2°C) for 24 hours with occasional shaking. Then, the mixtures were filtered through two layers of cheesecloth and centrifuged for 20 min at 10.000 rpm to remove particulate materials. Next, the purified extracts were adjusted to pH 6.8 with 1.0 M HCl. Finally, the extracts were stored in the refrigerator at 4°C to be used in the future.

### 2.3. Fungal Material

*Fusarium graminearum* (ITEM-6477) and* Fusarium sporotrichioides* (ITEM-692) were kept at the CREA-GB, Research Centre for Genomics and Bioinformatics, were multiplied, and were stored on potato dextrose agar (PDA) slants prior to use.

### 2.4. Effect of Plant Extracts on Mycelium Growth

The poisoned food method was used in the preliminary screening of aqueous extracts for their antifungal properties evaluation. First, the mycelia growths were evaluated in 60 mm Petri dishes filled with PDA solid medium amended with 10% and 20% aqueous extracts of each plant. Next, the center of each Petri dish was inoculated with 5 mm diameter disc of fungal mycelium, taken from pure culture (7 days old). Then, all inoculated dishes were incubated at 25°C for 6 days. After that, the radial mycelial growth was measured 6 days after inoculation. For each treatment, three replicates were maintained. Finally, the antifungal activity of each extract was calculated in terms of inhibition percentage of mycelia growth by using the following formula:

(1) $$\begin{array}{c} \%\text{ inhibition}=\left(dc-dt\right)/dc\times 100, \end{array}$$

where *dc* is the average increase in mycelia growth in control and *dt* is the average increase in mycelia growth in treated [19].

### 2.5. Phytochemical Screening of Plant Materials

The presence of polyphenol flavonoids, tannins, saponins, alkaloids, and steroids was tested using simple qualitative method as previously described [20, 21].

### 2.6. Statistical Analysis

Analysis of variance (ANOVA) was used for data analysis using CoStat-Statistics Software version 6.4. The significance of the differences among treated samples was evaluated using the least significant difference (LSD) test for multiple comparisons of the means of the growth diameter of mycelia. Each experiment has three replicates. Three determinations were conducted and the significance level for all measurements was considered at *P* < 0.05.

## 3. Results

### 3.1. Antifungal Activity of Plant Aqueous Extracts

The poisoned food technique, which involves both contacting between extracts and microorganisms and observing the growth of these, suggested that all the aqueous extracts under study exerted an inhibitory activity on Fusarium* graminearum* and* Fusarium sporotrichioides* mycelium growth.  Table 1 shows the impact of various treatments on fungal mycelium growth in comparison with nontreated control. A marked variability was observed at the two levels of the efficacy of the extracts and of the sensitivity of the two Fusaria strains. Analysis of variance showed highly significant differences (*P* < 0.001) between fungal strains and between treatments. On the contrast, the interaction between the fungi and treatments was not significant.

Table 2 shows the mycelium growth indexes of the two fungal strains during treatment. The highest growth speed was recorded for* Fusarium graminearum* in the presence of 10%* Euphorbia guyoniana* extract (speed of 1 mm/h), whereas the speed decreased to 0.28 mm/h at 20% concentration of* Asphodelus tenuifolius* extract.* Fusarium sporotrichioides* showed the fastest growth in the presence of 10%* Cotula cinerea* extract with 0.65 mm/h and the slowest speed in the presence of 20%* Euphorbia guyoniana* extract (with 0.30 mm/h).

**Figure 1:**
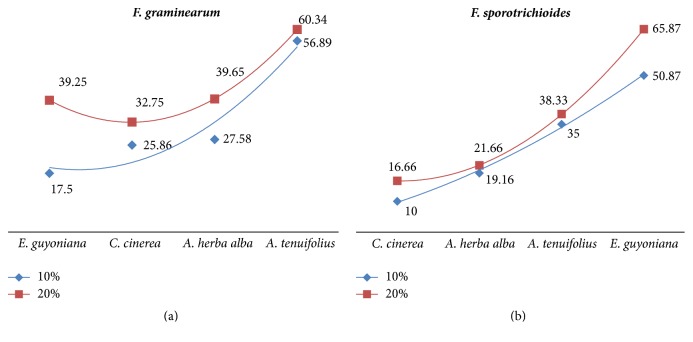
Percentage inhibition of* Fusarium graminearum* and* Fusarium sporotrichioides* mycelia growth treated with 10% and 20% plant extracts.

Figures 1(a) and 1(b) display the inhibition percentage of* in vitro* Fusaria growth in the presence of plant aqueous extracts.* Asphodelus tenuifolius* extract strongly inhibited* Fusarium graminearum* mycelium growth, with inhibition percentages of 56.89 and 60.34% at the concentrations 10 and 20% of aqueous extract, respectively. On the other hand,* Fusarium sporotrichioides* is inhibited by* Euphorbia guyoniana* extract: inhibition percentages of 50.87% and 65.87% were observed at concentrations of 10% and 20% aqueous extracts, respectively.

### 3.2. Phytochemical Characterization of Plant Extracts

The chemical characterization of the four plants showed the presence of polyphenols in the form of flavonoids and tannins, while anthocyanin was found only in* E. guyoniana*. The alkaloids that reacted positively overlooked in all tested plants, whereas steroids were noted in the* A. herba alba*, the* A. tenuifolius*, and* E. guyoniana*. Additionally, this study revealed the absence of both steroids and saponins in* C. cinerea* and* A. tenuifolius* extracts, respectively (Table 3).

## 4. Discussion

This study showed that all the aqueous extracts obtained from the aerial parts of* Artemisia herba alba, Cotula cinerea*,* Asphodelus tenuifolius*, and* Euphorbia guyoniana* have antimicrobial properties. Furthermore, marked variability has been observed for the* in vitro* sensitivity of the two phytopathogenic fungi tested, that is,* Fusarium graminearum* and* Fusarium sporotrichioides*, to various plant extracts.* Asphodelus tenuifolius* was found to be highly effective in controlling the growth of* Fusarium graminearum,* followed in terms of efficacy by* Artemisia herba alba* and Euphorbia* guyoniana* at the two concentrations used. The scale of potency of the four extracts in inhibiting the* Fusarium sporotrichioides* mycelial growth is as follows:* Euphorbia guyoniana > Asphodelus tenuifolius > Artemisia herba alba > Cotula cinerea.* The last plant extracts are shown to be the least effective one in reducing* in vitro* mycelium growth of both fungi. It is noteworthy that, among these tested plants,* C. cinerea* is the only plant that lacks steroids.* Euphorbia guyoniana* extract is particularly effective against* F. sporotrichioides* and is the only extract that contains anthocyanins. The other two plants, namely,* Asphodelus tenuifolius* and* Artemisia herba,* differ in terms of the presence/absence of saponins and are both effective in fungal growth inhibition. The antifungal effects of aqueous extracts of these plants* A. tenuifolius* and* E. guyoniana* can be attributed to the presence of different phytochemicals that can act alone or in synergy, as demonstrated by other studies [22–25]. It has been pointed out that there is a relationship between the antifungal activity of the extracts and its bioactive compounds [26]. In addition to this, the tannins isolated from the medicinal plants possess remarkable toxic activity against bacteria and fungi and they may assume pharmacological importance [27]. Furthermore, saponins are a special class of glycosides that have soapy characteristics and it is consider as active antifungal agents [28].

## 5. Conclusion

In conclusion, the aqueous extracts of some medicinal plants growing wild in the Northern Saharian environment of Algeria exhibited good antifungal activities and were capable of reducing growth of fungi responsible for alterations in wheat. These preliminary results, obtained from* in vitro* experiments, may be supplemented by other more comprehensive studies* in vivo*, both in controlled greenhouse conditions and in open field to practically evaluate the use of these extract in the frame of an Integrated Pest Management System. To date, in fact, a limited number of commercially developed natural plant compounds which are available for the agricultural industry were reviewed. While some of these compounds have antimicrobial activity, some others can act even as elicitors of systemic acquired resistance (SAR) through the activation of plant defense mechanisms. It is therefore of great interest even to deepen our knowledge about the molecular mechanisms of action of Algerian plant aqueous extracts not only against microorganisms, but even on plant biology. Subsequently, this can help researchers to look for new biostimulatory agents.

## Figures and Tables

**Table 1:** Effects of 10% and 20% concentrations of aqueous extracts of different plants on growth of *Fusarium graminearum *and* F. sporotrichioides*.

Treatment		Mycelia Diameter (mm)		Means
		*F. graminearum*	*F. sporotrichioides*	
Control	0.0%	58^a^ ± 0.28	60^a^ ± 0.00	59^a^
*Artemisia herba alba*	10.0%	42^c^ ± 0.57	48.16^d^ ± 0.44	45.08^c^
	20.0%	35^e^ ± 0.50	47^d^ ± 0.57	41^d^
*Cotula cinerea*	10.0%	43.66^c^ ± 0.57	54^b^ ± 0.57	48.83^b^
	20.0%	39^d^ ± 0.57	50^b^ ± 0.57	44.5^c^
*Asphodelus tenuifolius*	10.0%	25^f^ ± 0.57	39^e^ ± 0.57	32^e^
	20.0%	23^g^ ± 0.57	37^f^ ± 0.57	30^f^
*Euphorbia guyoniana*	10.0%	47.85^b^ ± 0.00	39.3^e^ ± 0.33	43.57^c^
	20.0%	35.23^e^ ± 0.2	27.3^g^ ± 0.88	31.26^e^
Means	—	38.74^b^	44.64^a^	—

Data reported are the mean diameters expressed in mm, including the inoculation discs of 5 mm. Values are presented as mean ± SEM. Different letters indicate significant differences (LSD test; *P* < 0.05).

**Table 2:** Effects of 10% and 20% concentrations of aqueous extracts of different plants on *Fusarium graminearum *and* F. sporotrichioides *mycelial growth index.

Treatment		Mycelia growth index (mm/h)		Means
		*F. graminearum*	*F. sporotrichioides*	
Control	0%	0.65^e^ ± 0.028	0.70^e^ ± 0.057	0.675^a^
*Artemisia herba alba*	10%	0.47^cd^ ± 0.005	0.57^cd^ ± 0.005	0.52^bc^
	20%	0.40^f^ ± 0.028	0.53^cd^ ± 0.033	0.46^c^
*Cotula cinerea*	10%	0.51^d^ ± 0.005	0.65^de^ ± 0.028	0.58^b^
	20%	0.46^e^ ± 0.005	0.61^de^ ± 0.01	0.535^b^
*Asphodelus tenuifolius*	10%	0.31^a^ ± 0.005	0.48^bc^ ± 0.011	0.395^d^
	20%	0.28^a^ ± 0.008	0.50^c^ ± 0.057	0.393^d^
*Euphorbia guyoniana*	10%	1^g^ ± 0.00	0.40^b^ ± 0.05	0.7^a^
	20%	0.90^f^ ± 0.00	0.30^a^ ± 0.057	0.65^a^
Means	—	**0.55** ^a^	**0.53** ^a^	—

The radial mycelial growth was measured 6 days after inoculation (the data reported are the mean diameters expressed in mm, including the inoculation discs of 5 mm). Values are represented as mean ± SEM. Different letters indicate significant differences (LSD test; *P* < 0.05).

**Table 3:** Phytochemical constituents on the aqueous extracts of tested plants.

Chemical group	Aqueous extract			
	*A. herba alba*	*C. cinerea*	*A. tenuifolius*	*E guyoniana*
*Polyphenols*				
Tannins	+	+	+	+
Anthocyanins	−	−	−	**+**
Flavonoids	+	+	+	+
Saponins	+	+	−	+
Alkaloids	+	+	+	+
Steroids	+	−	+	+

+ = presence and − = absence.

## References

[1] Satish S., Mohana D. C., Ranhavendra M. P., Raveesha K. A. (2007). Antifungal activity of some plant extracts against important seed borne pathogens of *Aspergillus* sp. *Journal of Agricultural Technology*.

[2] Nielsen P. V., Rios R. (2000). Inhibition of fungal growth on bread by volatile components from spices and herbs, and the possible application in active packaging, with special emphasis on mustard essential oil. *International Journal of Food Microbiology*.

[3] Bhatnagar D., mcCormick S. P. (1988). The inhibitory effect of neem (*Azadirachta indica*) leaf extracts on aflatoxin synthesis in *Aspergillus parasiticus*. *Journal of the American Oil Chemists' Society*.

[4] Terzi V., Tumino G., Stanca A. M., Morcia C. (2014). Reducing the incidence of cereal head infection and mycotoxins in small grain cereal species. *Journal of Cereal Science*.

[5] Varma J., Dubey N. K. (1999). Prospectives of botanical and microbial products as pesticides of tomorrow. *Current Science*.

[6] Pretorius J. C., Van der Watt E., Dubey N. K. (2011). Natural products from plants: commercial prospects in terms of antimicrobial, herbicidal and bio-stimulatory activities in an integrated pest management system. *Natural Products in Plant Pest Management*.

[7] Tegegne G., Pretorius J. C., Swart W. J. (2008). Antifungal properties of *Agapanthus africanus* L. extracts against plant pathogens. *Crop Protection*.

[8] Simin N., Seyyed A. E., Abolfz S., Mahmoud S. S., Yeganeh S. S. Antifungal Activity of Spearmint (*Mentha Spicata* L.) Essential Oil on *Fusarium oxysporum* f. sp. radicis cucumerinum the Causal Agent of Stem and Crown Rot of Greenhouse Cucumber in Yazd, Iran.

[9] Feeny P., Wallace J., Mansell R. (1976). Plant apparency and chemical defense in biochemical interaction between plants and insects. *Recent Advances in Phytochemistry*.

[10] Terzi V., Morcia C., Faccioli P., Valè G., Tacconi G., Malnati M. (2007). In vitro antifungal activity of the tea tree (*Melaleuca alternifolia*) essential oil and its major components against plant pathogens. *Letters in Applied Microbiology*.

[11] Parekh J., Karathia N., Chanda S. (2006). Evaluation of antibacterial activity and phytochemical analysis of *Bauhinia variegata* L. bark. *African Journal of Biomedical Research*.

[12] Aliero A. A., Afolayan A. J. (2006). Antimicrobial activity of *Solanum tomentosum*. *African Journal of Biomedical Research*.

[13] Buwa L. V., van Staden J. (2006). Antibacterial and antifungal activity of traditional medicinal plants used against venereal diseases in South Africa. *Journal of Ethnopharmacology*.

[14] Ergene A., Guler P., Tan S., Mirici S., Hamzaoglu E., Duran A. (2006). Antibacterial and antifungal activity of *Heracleum sphondylium* subsp. *Artvinense*. *African Journal of Biomedical Research*.

[15] Mohana D. C., Raveesha K. A., Rai K. M. L. (2008). Herbal remedies for the management of seed-borne fungal pathogens by an edible plant *Decalepis hamiltonii* (Wight & Arn). *Archives of Phytopathology and Plant Protection*.

[16] Mohamed A. E.-H. H., El-Sayed M. A., Hegazy M. E., Helaly S. E., Esmail A. M., Mohamed N. S. (2010). Chemical constituents and biological activities of *Artemisia herba-alba*. *Records of Natural Products*.

[17] Eddine L. S., Segni L., Ridha O. M. (2015). In vitro assays of the antibacterial and antioxidant properties of extracts from *Asphodelus tenuifolius* Cav and its main constituents: a comparative study. *International Journal of Pharmaceutical and Clinical Research*.

[18] Djellouli M., Moussaoui A., Benmehdi H., Ziane L., Belabbes A., Badraoui M. (2013). Ethnopharmacological study and phytochemical screening of three plants (Asteraceae family) from the region of south west Algeria. *Asian Journal of Natural & Applied Sciences*.

[19] Singh J., Tripathi N. N. (1999). Inhibition of storage fungi of blackgram (*Vigna mungo*) by some essential oils. *Flavour and Fragrance Journal*.

[20] Dohou N., Yamani K., Tahrouch S., Hassani l. M. I., Badoc A., Gmira N. (2003). Screeninig phyto-chimique d’une endémique ibéro-marocaine, *Thymelaea lythroides*. *Bulletin de la Société de Pharmacie de Bordeaux*.

[21] Edeoga H. O., Okwu D. E., Mbaebie B. O. (2005). Phytochemical constituents of some Nigerian medicinal plants. *African Journal of Biotechnology*.

[22] Field B., Jordán F., Osbourn A. (2006). First encounters—deployment of defence-related natural products by plants. *New Phytologist*.

[23] Rongai D., Milano F., Sciò E. (2012). Inhibitory effect of plant extracts on conidial germination of the phytopathogenic fungus *Fusarium oxysporum*. *American Journal of Plant Sciences*.

[24] Elad Y. (1991). Multiple resistance to benzimidazoles dicarboximides and diethofencarb in field isolates of *Bobytis cinerea* in Israel. *Plant Pathology Journal*.

[25] Giordani R., Hadef Y., Kaloustian J. (2008). Compositions and antifungal activities of essential oils of some Algerian aromatic plants. *Fitoterapia*.

[26] Abdelghani S. B., Weaver L., Zidan Z. H., Hussein M. A., Keevil C. W., Brown R. C. D. (2008). Microware-assisted synthesis and antimicrobial activities of flavonoid derivatives. *Bioorganic & Medicinal Chemistry Letters*.

[27] Banso A., Adeyemo S. O. (2007). Evaluation of antibacterial properties of tannins isolated from *Dichrostachys cinerea*. *African Journal of Biotechnology*.

[28] Barile E., Bonanomi G., Antignani V. (2007). Saponins from *Allium minutiflorum* with antifungal activity. *Phytochemistry*.

